# Initiation of antidepressant medicines in Swedish patients diagnosed with COVID-19: a registry-based cohort study

**DOI:** 10.1007/s00127-026-03169-2

**Published:** 2026-07-17

**Authors:** Roberto Mužić, Fredrik Nyberg, Björn Wettermark, Mohammadhossein Hajiebrahimi

**Affiliations:** 1 Pharmacoepidemiology unit, Department of Pharmacy, Uppsala University, Uppsala, Sweden; 2 School of Public Health and Community Medicine, Institute of Medicine, Sahlgrenska Academy, University of Gothenburg, Gothenburg, Sweden

**Keywords:** Antidepressant, Drug unitization, COVID-19, Registry-based study, Sweden

## Abstract

**Purpose:**

While antidepressant therapy is a common treatment for post-COVID mental health symptoms, little is known about initiations of antidepressants in patients after COVID-19 and how sociodemographic factors modify this risk. This study investigated whether adults infected by COVID-19 were more likely to initiate antidepressant therapy compared with non-infected individuals, and examined the influence of socioeconomic factors on initiation patterns.

**Methods:**

We conducted a nationwide registry-based cohort study using data from the SCIFI-PEARL project, including all adults (≥ 18 years of age) in Sweden from 1 March 2020 to 31 March 2022. Exposed individuals had a COVID-19 infection, and unexposed individuals were matched 1:1 by sex and birth year. Stratified Cox regression models were used to estimate the risk of antidepressant (ATC: N06A) initiation, adjusting for socioeconomic status, and Charlson Comorbidity Index.

**Results:**

The cohort included 2,666,510 individuals (1,333,255 exposed and unexposed). Exposed individuals had a 16% higher risk of initiating antidepressants compared with unexposed individuals after full adjustment (HR: 1.16, 95% CI:1.15–1.17). The risk reduced by roughly 43% when we adjusted for post-index healthcare contacts as potential mediator. Higher risks were observed for unmarried (HR: 1.14, 95% CI:1.13–1.16), or unemployed/retired (HR 1.14, 95% CI: 1.13–1.16) individuals. Conversely, higher educational level and being born outside Sweden were associated with lower risk of initiation of antidepressant, (HR:0.70, 95% CI:0.68–0.71) and (HR:0.92, 95% CI: 0.91–0.94), respectively.

**Conclusions:**

COVID-19 exposure was associated with an increased risk of initiating antidepressant therapy, particularly among socioeconomically vulnerable groups. Differences by education and immigrant status suggest disparities in access to healthcare.

## Introduction

The COVID-19 pandemic has profoundly affected both physical and mental health worldwide. Emerging evidence indicates that the infection can lead to long-term psychological consequences, including depression and anxiety [1]. Research from Germany and the United States show that COVID-19 patients face a higher risk of depression and first-time psychiatric diagnoses compared to uninfected individuals or those with other health conditions [2, 3]. Other studies report elevated risks of severe psychiatric conditions across multiple follow-up periods after COVID-19 diagnosis relative to patients with acute respiratory distress syndrome or negative tests [4, 5]. These findings collectively suggest a consistent association between COVID-19 infection and adverse mental health outcomes.

Depression and anxiety are among the most common psychiatric sequelae following COVID-19. Based on the selection of the specific groups, depression and anxiety were prevalent among infected individuals, up to 45% and 47%, respectively, especially those with severe disease [6–8]. Treatment recommendations for depression and anxiety depend on severity: mild to moderate cases are often managed with non-pharmacological interventions, whereas moderate to severe cases typically require pharmacological therapy. Antidepressants are the primary pharmacological option, particularly in contexts where access to non-pharmacological treatments is limited [9, 10]. Antidepressant initiation may serve as a proxy for these outcomes, yet very few studies investigated how COVID-19 has influenced prescribing patterns [11]. Understanding these patterns is important as health systems risk underestimating the pandemic’s mental health burden and may miss opportunities for targeted interventions and resource planning.

Nevertheless, the relationship between COVID-19 infection and subsequent initiation of antidepressant therapy remains unclear. Factors such as sex, age, and socioeconomic status may further influence prescribing patterns of antidepressants, which have been investigated in some previous studies [12, 13], yet need more deep investigations. Addressing this gap is important to guide clinical care and inform public health planning. This study therefore aimed to investigate whether individuals exposed to COVID-19 were more likely to initiate antidepressant treatment compared to unexposed individuals. Additionally, we examined the influence of socioeconomic factors, including education level, marital status, employment status, and country of birth, on initiation of antidepressant therapy in patients with COVID-19.

## Materials and methods

### Study design

A registry-based cohort study using data from the SCIFI-PEARL (Swedish Covid-19 Investigation for Future Insights – a Population Epidemiology Approach using Register Linkage) [14] project was conducted in a Swedish setting.

### Data source

The study used data from the following register sources included in SCIFI_PEARL: SmiNet [15], a national dataset of notifiable diseases, to obtain data on positive PCR test results; the Swedish Prescribed Drug Register (SPDR) [16] for data of all anti-depressant drugs and other concomitant drugs dispensed in Sweden; the National Patient Register (NPR) [17] for data on different comorbidities of the study participants registered during hospitalizations or outpatient consultations in specialist care clinics; the LISA (Longitudinal integration database for Health Insurance and Labor Market Studies) register at Statistics Sweden [18] for data on sociodemographic characteristics; and the National Cause of Death Register [19] for data on the date and cause of death.

### Population

The study population consisted all adults (≥ 18 years of age) between 1 March 2020 and 31 March 2022 who were resident in Sweden.

### Exposed and unexposed groups

The exposed group was defined as individuals with a COVID-19 diagnosis, defined as a positive SARS-CoV-2 polymerase chain reaction (PCR) test and/or a clinical diagnosis. Individuals without any COVID-19 diagnosis and without a positive PCR test constituted the unexposed group. To minimize the potential impact of the COVID-19 disease severity on the association between exposure and outcome, we excluded all patients hospitalized within two weeks of their COVID-19 diagnosis. To ensure comparability, an individual who was unexposed (no prior or current COVID-19) on the index date (the date of the COVID-19 diagnosis for the exposed individual) was matched 1:1 to each exposed individual, on sex and year of birth, and assigned the same index date.

### Outcomes

The primary outcome of the study was the initiation of antidepressant medication classified according to the Anatomical Therapeutic Chemical (ATC) classification system code N06A, which includes five drug groups: non-selective monoamine reuptake inhibitors (N06AA), selective serotonin reuptake inhibitors (N06AB), non-selective monoamine oxidase inhibitors (N06AF), selective monoamine oxidase inhibitors type A (N06AG), and other antidepressants (N06AX). We excluded all participants with a history of using any type of antidepressant drugs within 12 months preceding the index date (wash-out period) to ensure inclusion of only naïve antidepressant users in the study. The wash-out window was adapted to the Swedish prescription regulations where all prescriptions are valid for dispensing up to a maximum of 365 days after the have been issued.

### Covariates

Other studied variables included sex (male/female) and age on the index date (18–44, 45–64, 65.74, 75–85 and ≥ 85), educational level (primary school (≤ 9 years), high school (10–12 years), and academic level (> 12 years)), marital status (married/ unmarried including never married, divorced or widows), employment status (employed or unemployed/retired), country of birth (Sweden, Nordic countries excluding Sweden, European Union with 28 members (EU28) countries excluding Nordic countries, and countries outside the EU28). The latest available data before or at the index date was used for all covariates. Moreover, medication history (excluding antidepressants), in the two years before the index date was categorized into: 0, 1–4, 5–9, 10–14, and ≥ 15 medications; hospital admission or specialists’ clinic visits in the two years prior to the index date was classified into: 0, 1–5, 6–10, 11–20, and ≥ 21 visits; and the Charlson Comorbidity Index (CCI) between 2015.01.01 and end of 2019.12.31, categorized as 0, 1–2, 3–4, and ≥ 5.

### Statistical analysis

A descriptive analysis was performed to summarize the baseline characteristics of the exposed and unexposed groups. Categorical variables were reported as frequencies and percentages, while numerical variables were described using means with standard deviations (SD), or medians with interquartile ranges (IQR) and full ranges, to capture both central tendency and variability. To assess the risk of initiating antidepressant treatment over time, stratified Cox regression models were used to estimate hazard ratios (HR), with 95% confidence intervals (95% CI), both unadjusted and adjusted. Participants were followed from the index date, until the earliest of: emigration, death, antidepressant initiation, or the study end date (December 31, 2023). Three models were estimated: Model 1: unadjusted; Model 2: adjusted for socioeconomic status, including education level, marital status, employment status, and country of birth; Model 3: additionally adjusted for the CCI. To demonstrate the differences, a crude cumulative proportion curve was used to show the change in the prescribing patterns over time up to 48 months after index date in the exposed group compared to unexposed group. We also performed two sensitivity analyses where we adjusted model 3 for the number of drugs apart from anti-depressants (sensitivity analysis 1); and for the number of comorbidities in the two years preceding the index date of the study (sensitivity analysis 2). Moreover, to investigate the potential mediating effect of post-index healthcare contacts on the risk of antidepressant initiation, we adjusted for the mediator in the regression models. Before adjustment, we first compared the extent of post-index healthcare contacts between the exposed and unexposed groups to assess whether differences in healthcare utilization might explain the observed association. We then adjusted the crude model (Model 1) for the number of post-index healthcare contacts (Mediation Model 1). We then further adjusted this model for socioeconomic status (SES) together with the number of post-index healthcare contacts (Mediation Model 2). All analyses were conducted using SAS version 9.4 SAS Institute Inc., Cary, NC, USA.

## Results

The study included a total of 2,666,510 individuals (1,333,255 individuals each for exposed and unexposed groups). Among participants, 52.5% were male. Analysis of baseline characteristics revealed several notable differences between the exposed and unexposed groups (Table 1). The exposed group had a slightly lower proportion of individuals with primary education (15.3% vs. 17.4% respectively). The exposed group also had a higher rate of married people (41.0% vs. 36.9%) but lower unemployment rate than the unexposed group (12.6% vs. 19.9%).

**Table 1:** Baseline characteristics of individuals over 18 years of age who had a COVID-19 diagnosis, along with their matched comparison cohort

	Total (No = 2,666,510)		Exposed (COVID-19) (No = 1,333,255)		Unexposed (No = 1,333,255)	
	No	%	No	%	No	%
**Sex**						
Male	1,400,256	52.5	700,128	52.5	700,128	52.5
Female	1,266,254	47.5	633,127	47.5	633,127	47.5
**Age at the index date**						
19–44	1,547,972	58.1	774,004	58.1	773,968	58.1
45–64	891,692	33.4	445,846	33.4	445,846	33.4
65–84	194,850	7.3	97,387	7.3	97,463	7.3
≥ 85	31,996	1.2	16,018	1.2	15,978	1.2
Median (IQR)	42 (30–53)		42 (30–53)		42 (30–53)	
Range	18–108		18–108		18–108	
**Educational level (Y)**						
Primary (≤ 9)	421,045	16.3	199,503	15.3	221,542	17.4
Secondary (10–12)	1,108,044	42.9	568,166	43.5	539,878	42.3
Tertiary (≥ 12)	1,053,700	40.8	538,922	41.3	514,778	40.3
Missing	83,721					
**Marital status**						
Married	1,037,363	39.0	547,147	41.1	490,216	36.9
Unmarried/Widow	1,625,894	61.0	785,701	58.9	840,193	63.2
Missing	3,253					
**Employment status**						
Employed	2,231,120	83.8	1,165,103	87.4	1,066,017	80.1
Unemployed/Retired	432,135	16.2	167,745	12.6	264,390	19.9
Missing	3255					
**Place of birth**						
Sweden	1,991,672	74.7	1,015,151	76.2	976,521	73.3
Nordics excl. Sweden	48,904	1.8	22,376	1.7	26,528	2.0
EU28 excl. Nordics	119,090	4.5	49,507	3.7	69,583	5.2
Outside Europe	506,540	19.0	246,116	18.5	260,424	19.5
Missing	304					
**Follow up time (month)**						
Mean (+ SD)	27.8 ± 7.8		27.7 ± 8.0		28.0 ± 7.5	
Median (+ SD)	25 (23–34)		24 (23–34)		25 (23–35)	
Time Rang	0–45		0–45		0–45	

For pre-index health-related characteristics of the participants, the exposed group exhibited a Charlson Comorbidity Index (CCI) > 0 in 6.6% of individuals compared with 5.8% in the unexposed group (Table 2). For medication usage, 38.7% of the exposed group and 33.2% of the unexposed group had a history of two or more prescription drugs (excluding antidepressants) dispensed in the two years prior to the index date. Similarly, healthcare contacts were higher in the exposed group, 26.3% and 22.3% among exposed and unexposed, respectively, for being admitted at hospital/visiting a specialist clinic ≥ 2 times during the two years preceding the index date.

**Table 2:** Historical health-related characteristics of individuals over 18 years of age, had a COVID-19 diagnosis in Sweden, along with their matched comparison cohort

	Total (No = 2,666,510)	Exposed (No = 1,333,255)	%	Unexposed (No = 1,333,255)	%
**Charlson comorbidity index**					
0	2,500,297	1,245,078	93.4	1,255,219	94.1
1–2	141,849	74,231	5.6	67,618	5.1
3–4	15,032	8719	0.7	6313	0.5
≥ 5	9332	5227	0.4	4105	0.3
**History of dispensed medications other than antidepressants prior to index date**					
0	514,983	251,107	18.8	263,876	19.8
1–4	1,502,900	731,432	54.9	771,468	57.9
5–9	461,984	244,840	18.4	217,144	16.3
10–14	129,853	71,539	5.4	58,314	4.4
≥ 15	56,790	34,337	2.6	22,453	1.7
**History of hospital visits in the two years preceding the index date (COVID-19 diagnosis /** **corresponding date)**					
0	1,364,588	649,379	48.7	715,209	53.6
1–5	1,043,468	544,853	40.9	498,615	37.4
6–10	172,332	91,894	6.9	80,438	6.0
11–20	68,150	36,864	2.8	31,286	2.4
≥ 21	17,972	10,265	0.8	7707	0.6

**Fig. 1:**
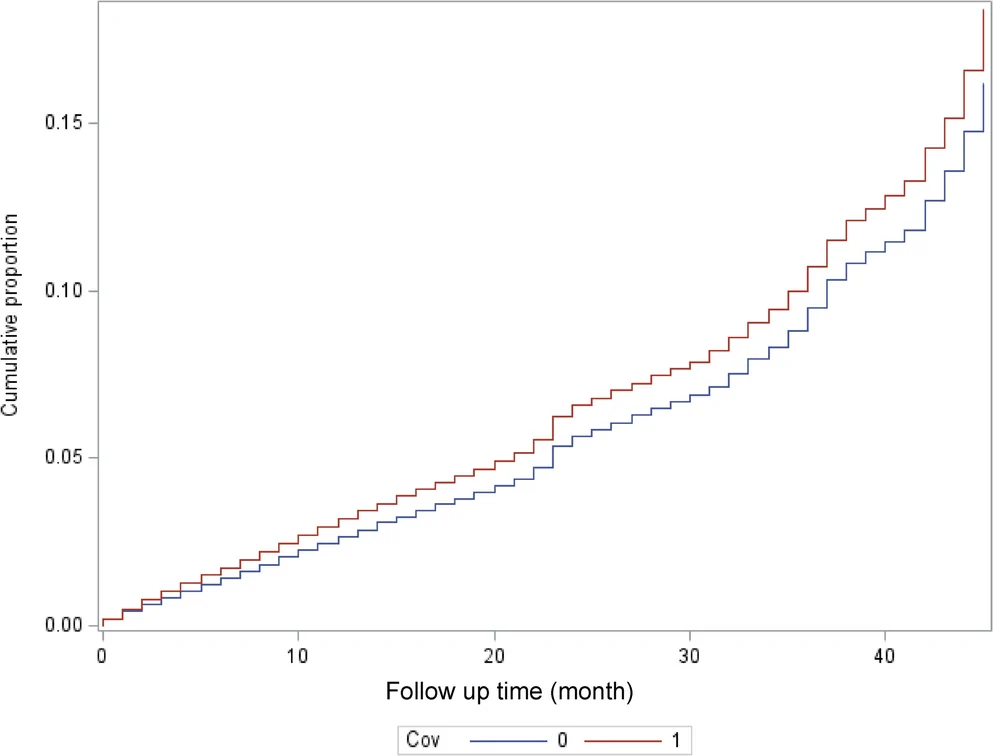
Crude cumulative proportion curve for initiation of new antidepressant drugs in patients with a COVID-19 diagnosis (Red line) compared to patients without a diagnosis (Blue line)

The unadjusted HR for antidepressants medicine initiation indicated 16% higher risk for individuals exposed to COVID-19 compared to those unexposed (HR: 1.16, 95% CI: 1.15–1.17) (Model 1) (Fig. 1; Table 3). The results remained almost the same after adjustment for socioeconomic status (Model 2) (HR:1.17 (95% CI: 1.16–1.18). In Model 3, adjusted for SES and Charlson Comorbidity Index didn’t change the result (HR:1.16, 95% CI: 1.15–1.17). Our first sensitivity analysis that adjusted model 2 for the history of non-antidepressant drugs in the two years preceding the index date showed a slightly lower HR of 1.12 (95% CI: 1.11–1.13). Adjusting model 2 for the history of comorbidities in the two years before the index date (Sensitivity analysis 2) made a small change in the results where HR was 1.14 (95% CI: 1.13–1.15). Comparison of post-index healthcare contacts, considered as a potential mediating factor, revealed a difference of 7% between the exposed and unexposed groups (64.3% vs. 57.1% with post-index healthcare contacts). After adjusting Model 1 for the number of post-index healthcare contacts (Mediation Model 1), the hazard ratio decreased to 1.09 (95% CI: 1.08–1.10). Further adjustment for socioeconomic status (SES) in addition to healthcare contacts, yielded similar results (HR: 1.10, 95% CI: 1.09–1.11) in Mediation Model 2.

**Table 3:** Hazard ratios (HR) and 95% confidence intervals (95%CI) for initiation of antidepressant treatment following exposure (COVID-19 diagnosis and/or positive test)

Models	HR	95% CI
**Model 1**: Unadjusted	1.16	1.15–1.17
**Model 2**: Adjusted for socioeconomic status	1.17	1.16–1.18
**Model 3**: Adjusted for Model 2 and Charlson Comorbidity Index	1.16	1.15–1.17
**Sensitivity analysis 1**: Adjusted for Model 2 and history of medicines consumption except antidepressants in 2 years preceding the index date	1.12	1.11–1.13
**Sensitivity analysis 2**: Adjusted for Model 2 and number of comorbidities in 2 years preceding the index date	1.14	1.13–1.15
**Mediation model 1**: Adjusted model 1 for the number of healthcare contacts after the index date	1.09	1.08–1.10
**Mediation model 2**: Adjusted Model 2 for the number of healthcare contacts after the index date	1.10	1.09–1.11

Several socioeconomic and health-related differences in initiation remained after adjustment (Table 4). Unmarried individuals and unemployed/retired individuals had a higher risk, with HRs of 1.14 (95% CI: 1.13–1.16) and 1.13 (95% CI: 1.11–1.16), respectively. Meanwhile, participants with higher educational level (HR:0.70, 95%CI:0.68–0.71) and who were born outside of Sweden (HR:0.92, 95%CI: 0.91–0.94) had a lower risk of initiation of the antidepressants.

**Table 4:** Hazard ratios (HR) and 95% confidence intervals (CI) for initiation of antidepressant treatment, adjusted for socioeconomic characteristics and Charlson Comorbidity Index

Variable	Adjusted HR*	95% CI
**COVID-19 Exposure**		
Unexposed	Reference	Reference
Exposed	1.16	1.15–1.17
**Education Level**		
Primary (≤ 9)	Reference	Reference
Secondary (10–12)	0.86	0.84–0.88
Tertiary (≥ 12)	0.70	0.68–0.71
**Marital Status**		
Married	Reference	Reference
Unmarried/Widow	1.14	1.13–1.16
**Employment Status**		
Employed	Reference	Reference
Unemployed/Retired	1.13	1.11–1.16
**Country of Birth**		
Sweden	Reference	Reference
Nordic excl. Sweden	0.97	0.92–1.02
EU28 excl. Nordic	0.93	0.90–0.97
Outside Europe	0.92	0.91–0.94
**Charlson Comorbidity Index (CCI)**		
0	Reference	Reference
1–2	1.46	1.42–1.50
3–4	1.62	1.50–1.75
≥ 5	1.53	1.39–1.69

*Each variable is included in the full adjusted model (model 3)

## Discussion

In this large cohort study, individuals exposed to COVID-19 had a 16% higher risk of initiating antidepressant treatment compared with unexposed individuals, after adjustment for socioeconomic factors and the Charlson Comorbidity Index (CCI). This risk decreased by approximately 43% in a mediation analysis that accounted for the number of post-index healthcare contact as a mediating variable. Higher risks of antidepressant initiation were also observed for being unmarried- and for unemployed/retired individuals. Conversely, individuals with higher educational attainment level and those born outside Sweden had a lower risk of starting antidepressant therapy.

The findings of this study are consistent with some previous studies reporting increased psychiatric morbidity following COVID-19 infection. For instance, Miyamori et al. observed an incidence rate ratio of 1.23 (95% CI 1.19–1.27) for antidepressant initiation one year after SARS-CoV-2 infection in a cohort of approximately 16 million participants with a median follow-up of 7 months [20]. In contrast, Nersesjan et al. reported a lower risk, with a hazard ratio of 0.85 (95% CI 0.81–0.90) for redemption of antidepressant prescriptions among individuals who tested positive compared with those who tested negative, based on a Danish cohort of about 4.1 million individuals followed for a mean of 1.83 years [21]. The inconsistent findings may partly reflect variations in population coverage and follow-up duration, with the Japanese study including a much larger cohort in absolute terms but representing a smaller share of the national population [20], whereas the Danish study covered a larger relative proportion of its population over a longer observation period [21].

Our results showed a reduction in risk in the mediation analysis. We assumed that the number of healthcare contacts would increase following a COVID-19 diagnosis, which in turn may lead to a higher likelihood of antidepressant prescriptions. We observed a 7% absolute difference between the exposed and unexposed groups in the number of healthcare contacts, indicating that this mediator accounted for roughly 43% of the total effect. These findings suggest that a substantial proportion of the effect of COVID-19 exposure operates through pathways independent of healthcare contacts. However, it is important to note that post-index healthcare contacts in this study were limited to outpatient specialist visits and hospital admissions. Data on primary care visits at national level were not available and therefore could not be included in the analysis. To our best knowledge, no previous studies have quantified the proportion of the association with antidepressant initiation that may be mediated by healthcare contact frequency after COVID-19 exposure.

The lower risk of initiation observed among non-Swedish born is in line with the “immigrant paradox,” primarily documented in the United States, where immigrant children and adolescents often demonstrate better mental health outcomes than their native-born peers, although these advantages may diminish over time [22, 23]. Immigrants also frequently face multiple barriers to mental healthcare, including language and cultural differences, limited awareness of available services, and stigma, contributing to underutilization of mental health resources [24]. Addressing such barriers is a priority for countries with substantial immigrant populations, including Sweden.

The elevated risk of antidepressant initiation among individuals with a higher number of dispensed medications likely reflects both polypharmacy and the higher prevalence of chronic conditions among older adults. Depression in adulthood often co-occurs with other medical conditions, increasing the likelihood of antidepressant prescribing. Adjusting for SES, CCI and the number of non-antidepressant drugs reduced the observed risk, suggesting that polypharmacy partially confounded the association between COVID-19 exposure and antidepressant initiation. Individuals with multiple health conditions or on several medications are more likely to receive additional prescriptions, sometimes leading to a prescribing cascade where new drugs are added to manage both existing illnesses and side effects from prior treatments [25]. Educational level was inversely associated with antidepressant initiation, supporting prior studies linking higher education to better mental health outcomes and greater access to non-pharmacological interventions, such as psychotherapy [26–28].

This study has several strengths and weaknesses worth noting. A key strength is the use of data from high-quality register data linked in the SCIFI-PEARL project, covering all adults in Sweden with capture of individuals with COVID-19 and selection of matched unexposed from the full population, ensuring a large, representative sample and high statistical power. Appropriate matching and adjustments and sensitivity analysis improve reliability. A wash-out period excludes prior antidepressant users, capturing new initiations. However, the Swedish prescribed drug register lacks data on the administered drugs at hospitals or in the specialist clinics which may lead to some misclassification and potentially underestimate the associations. The validity of the findings may also be limited by residual confounding and incomplete lifestyle data. Finally, generalizability may be restricted due to the predominantly Caucasian Swedish population.

## Conclusion

The risk of initiation of anti-depressant drugs increases by exposure to COVID-19 especially among socioeconomically vulnerable groups. Differences by education and immigrant status suggest disparities in care utilization. The findings highlight the mental health impact of COVID-19 and the need for targeted support for vulnerable groups.

## Data Availability

Data are available on reasonable request. Data are available on reasonable request from the corresponding author.
